# Protective role of *Clitoria ternatea* L. flower extract on methylglyoxal-induced protein glycation and oxidative damage to DNA

**DOI:** 10.1186/s12906-021-03255-9

**Published:** 2021-03-01

**Authors:** Poramin Chayaratanasin, Sirichai Adisakwattana, Thavaree Thilavech

**Affiliations:** 1 Program in Veterinary Biosciences, Faculty of Veterinary Sciences, Chulalongkorn University, Bangkok, 10330 Thailand; 2 Phytochemical and Functional Food Research Unit for Clinical Nutrition, Department of Nutrition and Dietetics, Faculty of Allied Health Sciences, Chulalongkorn University, Bangkok, 10330 Thailand; 3 Department of Food Chemistry, Faculty of Pharmacy, Mahidol University, Bangkok, 10400 Thailand

**Keywords:** *Clitoria ternatea*, Anthocyanins, Methylglyoxal, Protein glycation, Oxidative DNA damage

## Abstract

**Background:**

Methylglyoxal (MG) is a highly reactive dicarbonyl precursor for the formation of advanced glycation end products (AGEs) associated with age-related diseases, including diabetes and its complications. *Clitoria ternatea* L. flower has been reported to possess antioxidant and antiglycating properties. Evidence indicates that the extract of *Clitoria ternatea* L. flower inhibits fructose-induced protein glycation and oxidative damage to bovine serum albumin (BSA). However, there is no evidence to support the inhibitory effect of CTE against MG-mediated protein glycation and oxidative damage to protein and DNA. Therefore, the aim of the present study was to investigate whether *C. ternatea* flower extract (CTE) prevents MG-induced protein glycation and oxidative DNA damage.

**Methods:**

The formation of fluorescent AGEs in BSA was evaluated using spectrofluorometer. The protein carbonyl and thiol group content were used for detecting protein oxidation. DNA strand breakage in a glycation model comprising of MG, lysine and Cu^2+^ or a free radical generator 2,2′-azobis(2-methylpropionamidine) dihydrochloride (AAPH) systems was investigated using gel electrophoresis. Generation of superoxide anions and hydroxyl radicals in the MG/lysine system was assessed by the cytochrome *c* reduction assay and thiobarbituric acid reactive substances assay, respectively. High performance liquid chromatography (HPLC) was used to measure the MG-trapping ability.

**Results:**

In the BSA/MG system, CTE (0.25–1 mg/mL) significantly inhibited the formation of fluorescent AGEs and protein oxidation by reducing protein carbonyl content as well as preventing the protein thiol depletion. The concentration of CTE at 0.125–1 mg/mL prevented oxidative DNA cleavage in MG/lysine and AAPH systems associated with the inhibition of superoxide anion and hydroxyl radical formation. It also directly trapped MG in a concentration-dependent manner, ranging from 15 to 43%.

**Conclusions:**

The study findings suggest that the direct carbonyl trapping ability and the free radical scavenging activity of CTE are the underlying mechanisms responsible for the prevention of protein glycation and oxidative DNA damage.

**Supplementary Information:**

The online version contains supplementary material available at 10.1186/s12906-021-03255-9.

## Background

Advanced glycation end products (AGEs) are the products of non-enzymatic reaction between reducing sugars (such as glucose and fructose) and free amino groups in biological molecules [1]. AGE accumulation in the circulating blood and various tissues has been reported in patients with diabetes and plays a significant role in the development of further complications commonly associated with this disease such as nephropathy [2]. A body of evidence also shows that AGEs are linked to changes that occur naturally with aging and the development of age-related diseases such as Alzheimer’s disease, Parkinson’s disease, and renal dysfunction [3, 4]. Methylglyoxal (MG) has been recognized as a potent precursor of AGE formation [5]. MG is endogenously produced as a byproduct of carbohydrate, lipid and protein metabolism, glycolysis pathway in particular. In addition to being glycolytic intermediate, MG is also generated as an intermediate in the non-enzymatic glycation reaction. Under physiological conditions, MG is mainly degraded to D-lactate by glyoxalase enzymatic system [6]. In comparison with healthy subjects, higher MG levels are typically observed in patients with diabetes [7]. As a significant amount of dicarbonyl compounds is generated during glycation process, these intermediates are a predominant factor relating to acceleration of AGE production [8, 9]. The binding of AGEs to the receptor for AGEs (RAGE) stimulates the production of reactive oxygen species (ROS) and inflammatory cytokines, resulting in oxidative stress and inflammation [10]. Non-enzymatic crosslinking of MG with lysine or arginine residues of proteins during AGE formation also contributes to ROS generation including superoxide anions and hydroxyl radicals [11]. These highly toxic compounds trigger oxidative modification and subsequent damage to cellular components such as proteins [12] and DNA [13, 14]. Finding yielded by recent studies in this field indicate that ROS-mediated oxidative damage contributes to the pathogenesis associated with numerous chronic diseases such as cancer, atherosclerosis and cardiovascular disease [15].

Attenuation of MG accumulation and the reduction of its deleterious effects has been recognized as a potential target for therapeutic intervention. Consequently, the synthetic compounds demonstrating MG-trapping ability have received considerable research interest owing to their potential anti-glycation activity [16]. Aminoguanidine (AG), a well-known anti-glycating agent, was reported to inhibit AGE formation, which prevented the development of diabetic complications in animal models of diabetes [17]. Nevertheless, AG failed in the early phase of clinical trials due to its serious adverse effects, including myocardial infarction, congestive heart failure, atrial fibrillation, anemia and gastrointestinal disturbance [18, 19]. This has prompted research into plant-based foods as a mean of preventing and ameliorating AGE-mediated diabetic complications [20, 21].

In extant studies, anti-glycative inhibitors from natural sources have been shown to exhibit a preventive effect on AGE-associated diseases by scavenging free radicals and conjugating MG [22, 23]. Anthocyanins, natural pigments in fruits and vegetables, have been shown to exert beneficial effects such as antioxidant, anti-hyperglycemic and anti-glycation activity [24, 25]. *Clitoria ternatea* L. is an herbaceous plant from the Fabaceae family that has been used in traditional medicine for centuries. The major anthocyanins identified from *C. ternatea* flower are delphinidin-based ternatins and flavonal glycosides [26, 27]*.* Recently, *C. ternatea* flower (CTE) has been reported to suppress postprandial glycemic response in participants who consumed it with beverage containing sugar, which was ascribed to its inhibitory activity against intestinal α-glucosidase [28, 29]. In addition, CTE has been shown to exhibit anti-glycation activity against fructose-induced glycation and oxidation in bovine serum albumin (BSA) model [30], while also inhibiting adipogenesis in 3T3-L1 cells [27]. However, the effect of CTE on the prevention of MG-mediated protein glycation and oxidative damage to protein and DNA remains unknown. This gap in extant knowledge has motivated the present study, the aim of which was to examine the effect of CTE against MG-induced glycation and oxidation in BSA. As a part of this investigation, the effect of CTE on 2,2′-azobis(2-methylpropionamidine) dihydrochloride (AAPH) and MG/lysine-induced oxidative damage to DNA was also determined. Finally, it possible mechanisms of action were explored by examining the effect of CTE on the direct trapping of MG as well as on the generation of superoxide anions and hydroxyl radicals.

## Methods

### Chemicals and reagents

Methylglyoxal (MG) 40% solution, aminoguanidine (AG), bovine serum albumin (BSA), guanidine hydrochloride, L-cysteine, *o*-phenylenediamine (*ο*-PD), 5-methylquinoxaline (5-MQ), 2,2′-azobis(2-methylpropionamidine) dihydrochloride (AAPH), 2-deoxy-D-ribose, Cytochrome *c* from equine heart and 2-thiobarbituric acid (TBA) were obtained from Sigma (St. Louis, MO, USA). 2,4-dinitrophenylhydrazine (DNPH) was purchased from Ajax Finechem (Taren Point, NSW, Australia), whereas L-lysine hydrochloride was obtained from Himedia (L.B.S. Marg, MB, India). Finally, 5,5′-dithiobis(2-nitrobenzoic acid) (DTNB), trichloroacetic acid and methanol (gradient grade for liquid chromatography) were purchased from Merck (Darmstadt, Germany).

### Plant material

Dried *C. ternatea* flowers were obtained from the local herbal drug store in Bangkok, Thailand. The plant was authenticated at the Princess Sirindhorn Plant Herbarium, Plant Varieties Protection Division, Department of Agriculture, Thailand (Voucher specimen: BKU066793). The extraction process was based on a previously published method with some modifications [31]. Briefly, dried flowers were extracted with distilled water at 95 °C for 2 h after which the solution was filtered through Whatman 70 mm filter paper and further dried using spray dryer SD-100 (Eyela world, Tokyo Rikakikai Co., LTD, Japan). The spray drying was performed under the inlet temperature of 178 °C and the outlet temperature of 80 °C, with the blower set at 0.9 m^3^/min and atomizing at 90 kPa. The powder extract was kept in a laminate foil vacuum bag at − 20 °C until required for analysis. Total phenolic compounds in CTE determined at 53.0 ± 0.34 mg gallic acid equivalent/g dried extract, and total anthocyanin was 1.46 ± 0.04 mg cyanidin-3-glucoside equivalent/g dried extract [30]. According to our previous studies, delphinidin derivatives (such as delphinidin-3-*O*-(6-*O*-*p*-coumaryl) glucoside-pyruvic acid and delphinidin-3-*O*-glucoside) and flavonol glycosides (such as keamferol-3-*O*-rutinoside and quercetin-3-*O*-rutinoside) were identified as major bioactive compounds in CTE [27].

### Bovine serum albumin (BSA)-MG assay

Glycated BSA formation was performed in the line with previously adopted protocols with minor modifications [32]. In brief, the reaction mixture consisted of BSA (10 mg/mL) and MG (1 mM) in a 0.1 M phosphate buffered saline (PBS), pH 7.4, containing 0.02% sodium azide. CTE (0.25–1 mg/mL) or AG (1 mg/mL) was further added to the mixture and the resulting sample was incubated at 37 °C for 7 days. The formation of fluorescent AGEs in the reaction mixture was determined using a spectrofluorometer (Perkin Elmer, MA, USA) at the excitation and emission wavelengths of 355 and 460 nm, respectively. PBS was used instead of BSA as a blank for fluorescence intensity subtraction. The percentage inhibition was calculated according to following equation:

$$ \mathrm{Inhibition}\ \mathrm{of}\ \mathrm{fluorescent}\ \mathrm{AGEs}\ \left(\%\right)=\left[\left(\left(\mathrm{FC}-\mathrm{FCB}\right)-\left(\mathrm{FS}-\mathrm{FSB}\right)/\left(\mathrm{FC}-\mathrm{FCB}\right)\right)\right]\times 100 $$

where F_C_ denotes fluorescence intensity of MG with BSA, F_CB_ represents the fluorescence intensity of the blank, and F_S_ and F_SB_ are the fluorescence intensity of CTE or AG with BSA/MG and the blank, respectively.

### Determination of protein carbonyl content

Protein carbonyl content was determined using 2,4-dinitrophenylhydrazine (DNPH) reagent according the previously described protocol [32]. Briefly, glycated BSA (100 μL) was incubated with 10 mM DNPH in 2.5 M HCl (400 μL) for 1 h in dark with repeated mixing at 10 min intervals, and 500 μL trichloroacetic acid solution (20% w/v) was added to the mixture for protein precipitation. The reaction was performed on ice for 5 min and was centrifuged at 10,000 rpm at 4 °C for 10 min. The protein pellet was washed with 1 mL ethanol/ethyl acetate mixture (1:1 v/v) three times before being dissolved in 6 M guanidine hydrochloride (250 μL). Absorbance was measured at the 370 nm wavelength. The carbonyl content was calculated from the extinction coefficient for DNPH (*ε* = 22,000 M/cm) and the results were expressed as nmol carbonyls/mg protein.

### Determination of thiol group content

Thiol groups in glycated BSA were measured after 7 days of incubation according to the Ellman’s assay with slight modification [33]. Briefly, glycated BSA (70 μL) was mixed with 2.5 mM DTNB (130 μL) for 15 min at room temperature after which the absorbance was read at 412 nm. Free thiol concentration was calculated based on the standard L-cysteine curve.

### Analysis of DNA strand breaks

Oxidative damage to plasmid DNA was performed following a previously described protocol with some modifications [11]. Briefly, the reaction mixture (10 μL total volume) contained 0.25 μg pCU19 plasmid DNA (Thermo Scientific™, Carlsbad, CA, USA), 50 mM MG, 50 mM lysine, and 300 μM CuSO_4_ with or without CTE (0.125–1 mg/mL). In addition to the MG/lysine/Cu^2+^ system, a free-radical generator, AAPH (12.5 mM), was also used as an inducer of oxidative DNA cleavage. The reaction mixture was incubated for 3 h at 37 °C after which the reaction was stopped by freezing at − 20 °C for 90 min. The sample was subsequently assessed by 0.8% agarose gel electrophoresis in Tris-borate-EDTA (TBE) buffer. The band of plasmid DNA was stained by SYBR™ Safe DNA Gel Dye (Thermo Scientific™, CA, USA) before being visualized and photographed by Gel Doc imager (Syngene, UK) under UV light. The intensity of each band was quantified using GeneTools software (Syngene, UK). The relative amounts of supercoiled (SC) and open circular (OC) form of DNA were quantified by band intensity. The results were reported as relative percentage OC form of plasmid DNA using the expression below after subtracted by percentage OC form of DNA alone.

$$ \%\mathrm{open}\ \mathrm{circular}\ \left(\mathrm{OC}\right)\ \mathrm{form}=\frac{\mathrm{Intensity}\ \mathrm{of}\ \mathrm{OC}\ \mathrm{form}\ }{\mathrm{Intensity}\ \mathrm{of}\ \mathrm{SC}\ \mathrm{form}+\mathrm{OC}\ \mathrm{form}} \times 100 $$

### Determination of superoxide anion

Superoxide anion was determined by the cytochrome *c* reduction assay as previously described [34]. Briefly, the reaction mixture contained 20 mM MG and 20 mM lysine with or without CTE (0.125–1 mg/mL). Cytochrome *c* was then added to the reaction mixture to yield a final concentration at 10 μM. The cytochrome *c* reduction rate was measured at 10 min intervals as an increase in absorbance at 550 nm and 37 °C for over a 60 min period. The results were expressed as the amount of reduced cytochrome *c* (nmol/mL) calculated using the molar extinction coefficient of reduced cytochrome *c* at 550 nm (*ε* = 27,700 M/cm).

### Determination of hydroxyl radical

Presence of hydroxyl radicals was detected using thiobarbituric acid reactive 2-deoxy-D-ribose oxidation products [34]. For this purpose, MG (50 mM) was incubated with lysine (50 mM) and 2-deoxy-D-ribose (20 μM) in the absence or presence of CTE (0.125–1 mg/mL) at 37 °C for 3 h. Subsequently, 2.8% (w/v) trichloroacetic acid (200 μL) and 1% (w/v) thiobarbituric acid (200 μL) solutions were added to the assay mixture, which was then heated at 100 °C for 10 min followed by cooling down to room temperature. The degradation products of 2-deoxy-D-ribose by hydroxyl radicals were measured absorbance at wavelength of 532 nm. Finally, the generation of hydroxyl radicals was expressed as the level of thiobarbituric acid reactive substances (TBARS), which was calculated using the malondialdehyde standard curve.

### Determination of MG-trapping ability

CTE (0.25–1 mg/mL) or AG (1 mg/mL) was incubated with 1 mM MG in 0.1 M PBS (pH 7.4) at 37 °C. After 24 h of incubation, 20 mM *o*-phenylenediamine (*o*-PD) was added to the reaction mixture and was kept at room temperature for 30 min to convert remaining MG into 2-methylquinoxaline (2-MQ). MG quantification, which was based on the level of 2-MQ, was performed through high-performance liquid chromatography (HPLC) following a previously described protocol [35]. For this purpose, HPLC equipped with a LC-10 AD pump, SPD-10A UV-Vis detector and LC-Solution software (Shimadzu Corp., Kyoto, Japan) was utilized. A C18 (Inertsil ODS 3V) column (250 × 4.6 mm i.d.; 5 μm particle size) was used as stationary phase. The mobile phase of HPLC system composed of HPLC grade water and methanol (50:50, v/v). The flow rate and injection volume were 1.2 mL/min and 10 μL, respectively. The total running time was 15 min and the absorbance was finally recorded at the wavelength of 315 nm. 5-methylquinoxaline (5-MQ) in methanol was used as internal standard at a final concentration of 0.06% (v/v) and the percentage of MG reduction was calculated using the equation below:

$$ \%\mathrm{MG}\ \mathrm{trapping}\ \mathrm{capacity}=\frac{\left(\mathrm{Amount}\ \mathrm{of}\ \mathrm{MG}\ \mathrm{in}\ \mathrm{control}-\mathrm{Amount}\ \mathrm{of}\ \mathrm{MG}\ \mathrm{in}\ \mathrm{sample}\right)\ }{\mathrm{Amount}\ \mathrm{of}\ \mathrm{MG}\ \mathrm{in}\ \mathrm{control}}\times 100 $$

### Statistical analysis

Analysis results were expressed as means±standard error of means (SEM) for each experimental group (*n* = 3). Statistical significance was analyzed by One-Way ANOVA (SPSS version 18 for windows, SPSS Inc., Chicago, IL, USA), whereas differences between groups were assessed by conducting a Duncan’s post hoc test, whereby *p* ≤ 0.05 was considered as statistically significant. The resulting graphs were generated using Sigma Plot (version 12; Systat Software Inc., San Jose, CA, USA).

## Results

### The effect of CTE on the formation of fluorescent AGEs

**Fig. 1:**
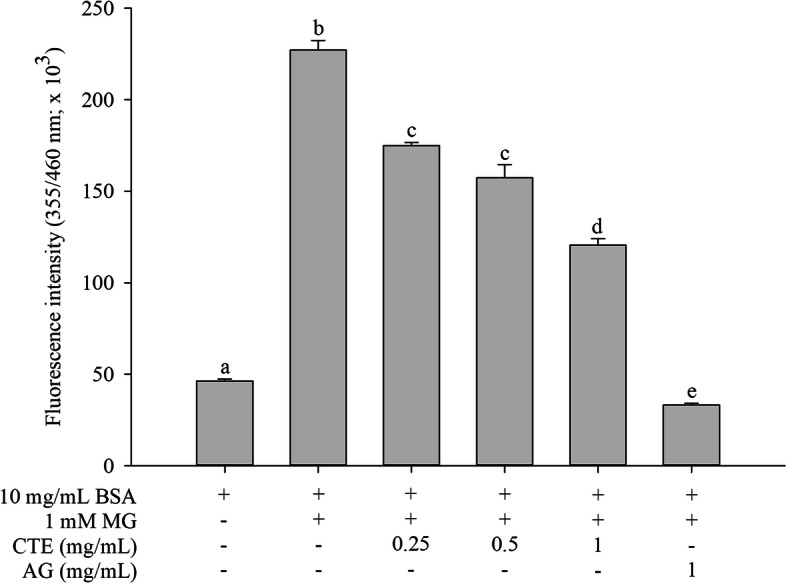
Effect of *Clitoria ternatea* flower extract (CTE, 0.25–1 mg/mL) and aminoguanidine (AG, 1 mg/mL) on fluorescent AGE formation in the BSA/methylglyoxal (MG) system. Each value represents the mean ± SEM (*n* = 3). Means with different letters are significant different (*p* < 0.05)

BSA incubation with MG caused a 4.5-fold increase in the formation of fluorescent AGEs when compared to BSA alone and this different was statistically significant (Fig. 1). The MG-induced BSA glycation was decreased as a concentration-dependent manner when CTE at 0.25–1 mg/mL concentrations was added. The highest CTE concentration (1 mg/mL) yielded the greatest reduction (46%) in the formation of fluorescent AGEs. However, AG produced a more potent reduction in fluorescent AGEs than CTE at equal concentration (1 mg/mL).

### The effect of CTE on protein oxidation

**Fig. 2:**
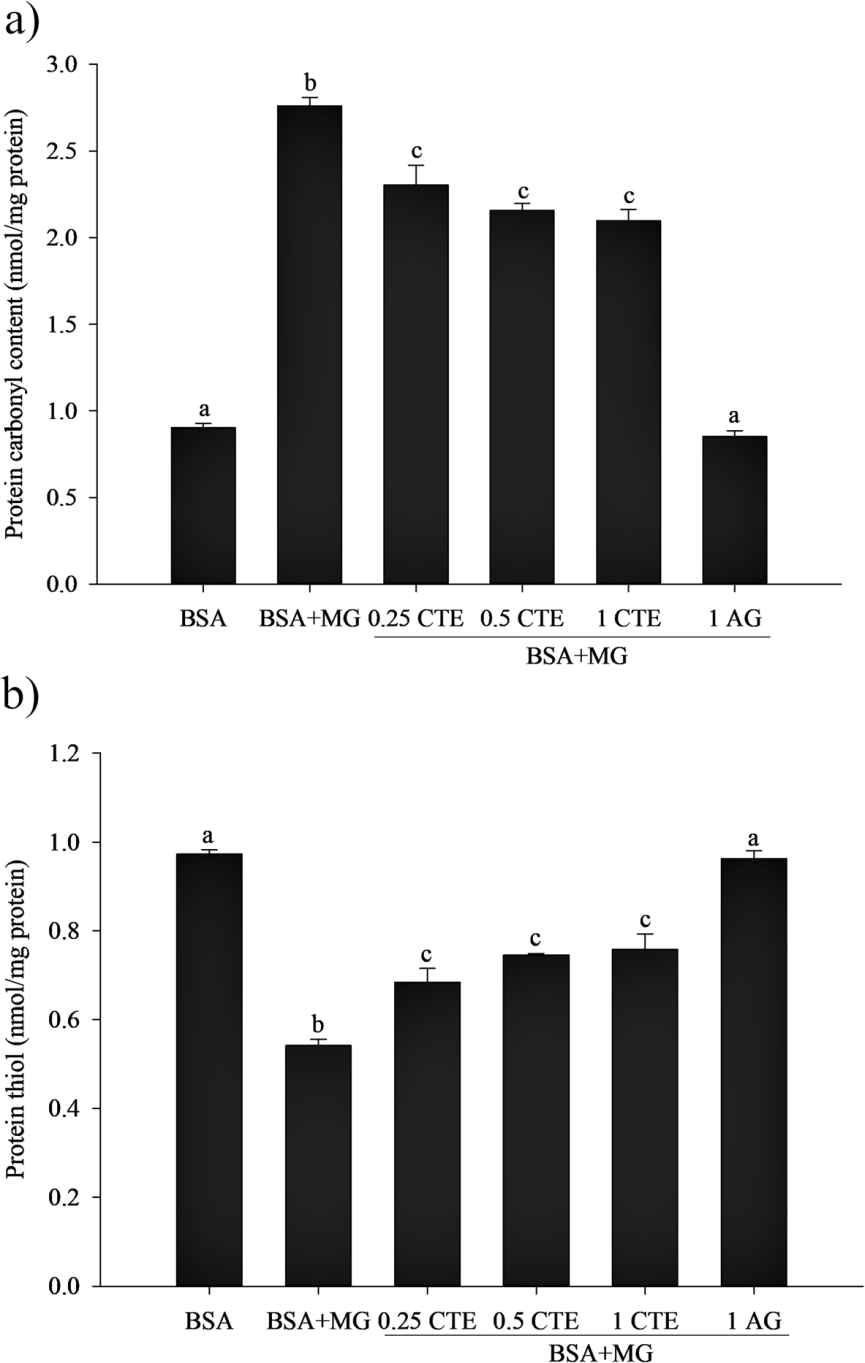
Effect of *Clitoria ternatea* flower extract (CTE, 0.25–1 mg/mL) and aminoguanidine (AG, 1 mg/mL) on the level of protein carbonyl **a** and protein thiol **b** in the BSA/methylglyoxal (MG) system. Each value represents the mean ± SEM (*n* = 3). Means with different letters are significant different (*p* < 0.05)

As shown in Fig. 2a, MG resulted in a 3-fold increase of carbonyl content in BSA after 7 days of incubation. Moreover, CTE (0.25–1 mg/mL) reduced protein carbonyl content in glycated BSA (*p* < 0.05). A 22% reduction of protein carbonyl content was detected in the BSA/MG system with CTE (1 mg/mL). In this reaction, AG (1 mg/mL) produced a greater inhibition percentage (69%) than CTE at the same concentration. The addition of AG reduced the level of protein carbonyl content which was a similar amount as BSA alone.

At the final (day 7) of incubation, BSA oxidation mediated by MG resulted in the depletion of protein thiol groups by 56% (Fig. 2b). Interestingly, the reduction of thiol groups was attenuated when CTE (0.25–1 mg/mL) was added into the BSA/MG system (*p* < 0.05). Compared to BSA/MG, CTE (1 mg/mL) arrested the depletion of thiol groups by 23%. At the same concentration (1 mg/mL), AG was 44% more effective in preserving protein thiol group than CTE, and the amount of protein thiol group in the BSA/MG system with AG was similar to that observed by BSA alone.

### The effect of CTE on oxidative DNA damage

**Fig. 3:**
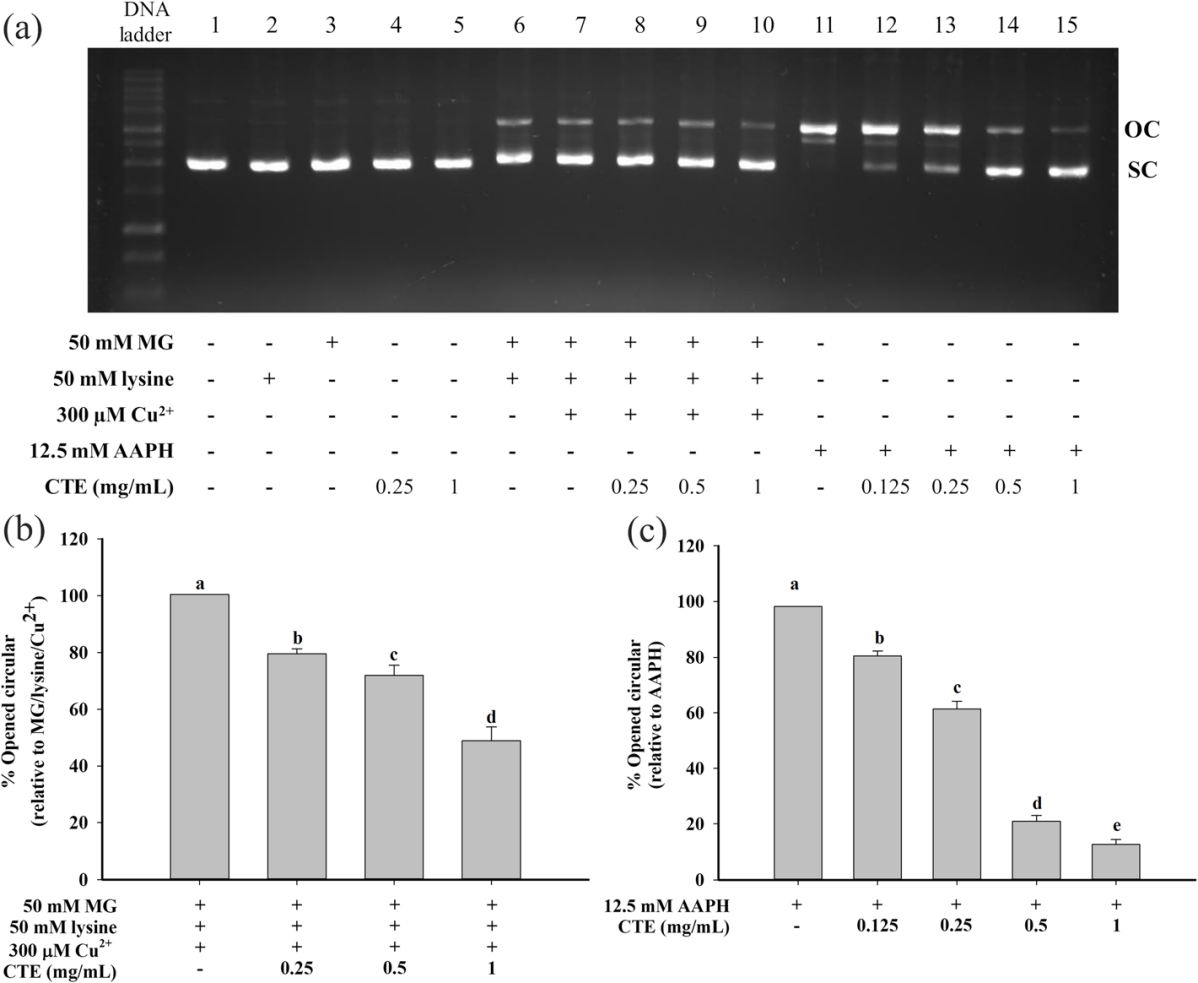
Effect of *Clitoria ternatea* flower extract (CTE) on DNA strand breakage in methylglyoxal (MG)/lysine and 2,2′-azobis(2-methylpropionamidine) dihydrochloride (AAPH) system. Supercoiled form (SC) and opened circular form (OC) represented as the major band and damaged plasmid DNA, respectively. Plasmid DNA (0.25 μg) was incubated with the following: **a** Lane 1, DNA alone; Lane 2, 50 mM MG; Lane 3, 50 mM lysine; Lane 4, 0.25 mg/mL CTE; Lane 5, 1 mg/mL CTE; Lane 6, MG + lysine; Lane 7, MG + lysine+Cu^2+^; Lane 8, MG + lysine+Cu^2+^+ 0.25 mg/mL CTE; Lane 9, MG + lysine+Cu^2+^+ 0.5 mg/mL CTE; Lane 10, MG + lysine+Cu^2+^+ 1 mg/mL CTE; Lane 11, 12.5 mM AAPH; Lane 12, AAPH+ 0.125 mg/mL CTE; Lane 13, AAPH+ 0.25 mg/mL CTE; Lane 14, AAPH+ 0.5 mg/mL CTE; Lane 15, AAPH+ 1 mg/mL CTE. The percentage of open circular (OC) form of plasmid DNA in **b** MG/lysine- and **c** AAPH-mediated oxidative strand breakage of plasmid DNA. Each value represents the mean ± SEM (*n* = 3). Means with different letters are significant different (*p* < 0.05)

The addition of MG, lysine or CTE (at the highest concentration of 1 mg/mL) alone did not cause the breakage of SC form, indicating that no damage to the plasmid DNA occurred (Fig. 3a). The incubation of plasmid DNA in MG/lysine/Cu^2+^ system markedly induced the breakage of SC form. As a result, OC band intensity increased when compared to untreated DNA (Fig. 3a). This damage was attenuated by CTE (8–53%) in a concentration dependent manner (0.25–1 mg/mL), as shown in Fig. 3b.

Similar pattern of DNA cleavage to the MG/lysine system was obtained by a free-radical mediator AAPH (Fig. 3a). The presence of CTE (0.125–1 mg/mL) in the system markedly reduced (16–88%) the OC band intensity, as shown in Fig. 3c.

### The effect of CTE on MG-induced generation of superoxide anions and hydroxyl radicals

**Fig. 4:**
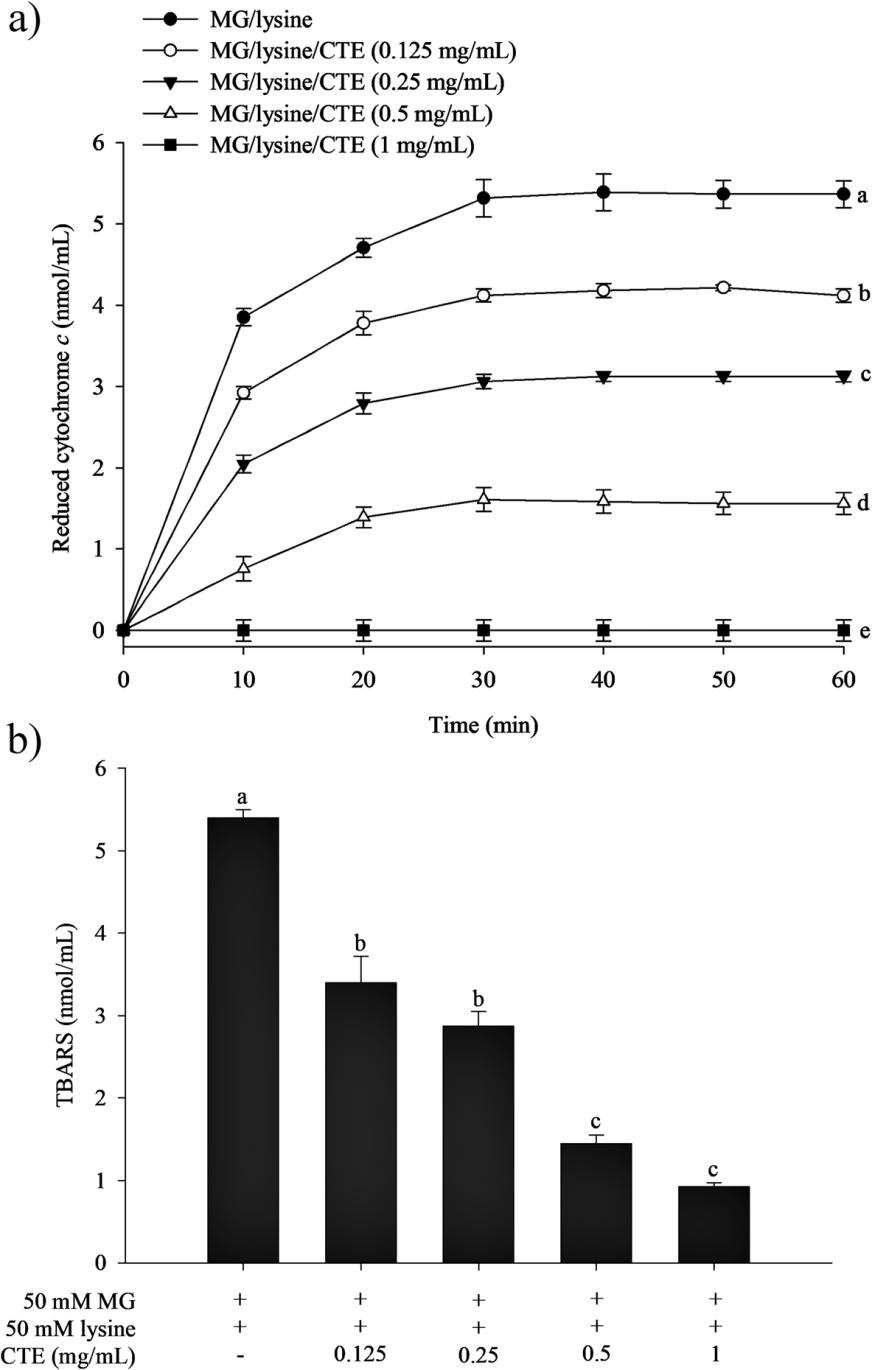
Effect of *Clitoria ternatea* flower extract (CTE) on the generation of superoxide anion as represented by the level of reduced cytochrome *c* **a** and hydroxyl radicals as represented by the level of thiobarbituric acid substances (TBARS) **b** in methylglyoxal (MG)/lysine-induced glycation. Each value represents the mean ± SEM (*n* = 3). Means with different letters are significant different (*p* < 0.05)

The amount of the reduced form of cytochrome *c* was increased in the MG/lysine system over the 7-day incubation period, indicating an increase in the production of superoxide anions (Fig. 4a). After 60 min, the reduced cytochrome *c* level was 5.42 ± 0.15 nmol/mL. CTE (0.125–1 mg/mL) suppressed the generation of superoxide anions in a concentration-dependent manner, ranging from 23 to 99% (*p* < 0.05). The significant inhibitory effect by CTE was observed after 10 min of incubation.

Figure 4b demonstrates an increase in the amount of TBARS mediated by the MG/lysine system, indicating increased hydroxyl radical production. Addition of CTE (0.125–1 mg/mL) was able to suppress the generation of hydroxyl radicals (*p* < 0.05).

### The MG trapping ability

**Fig. 5:**
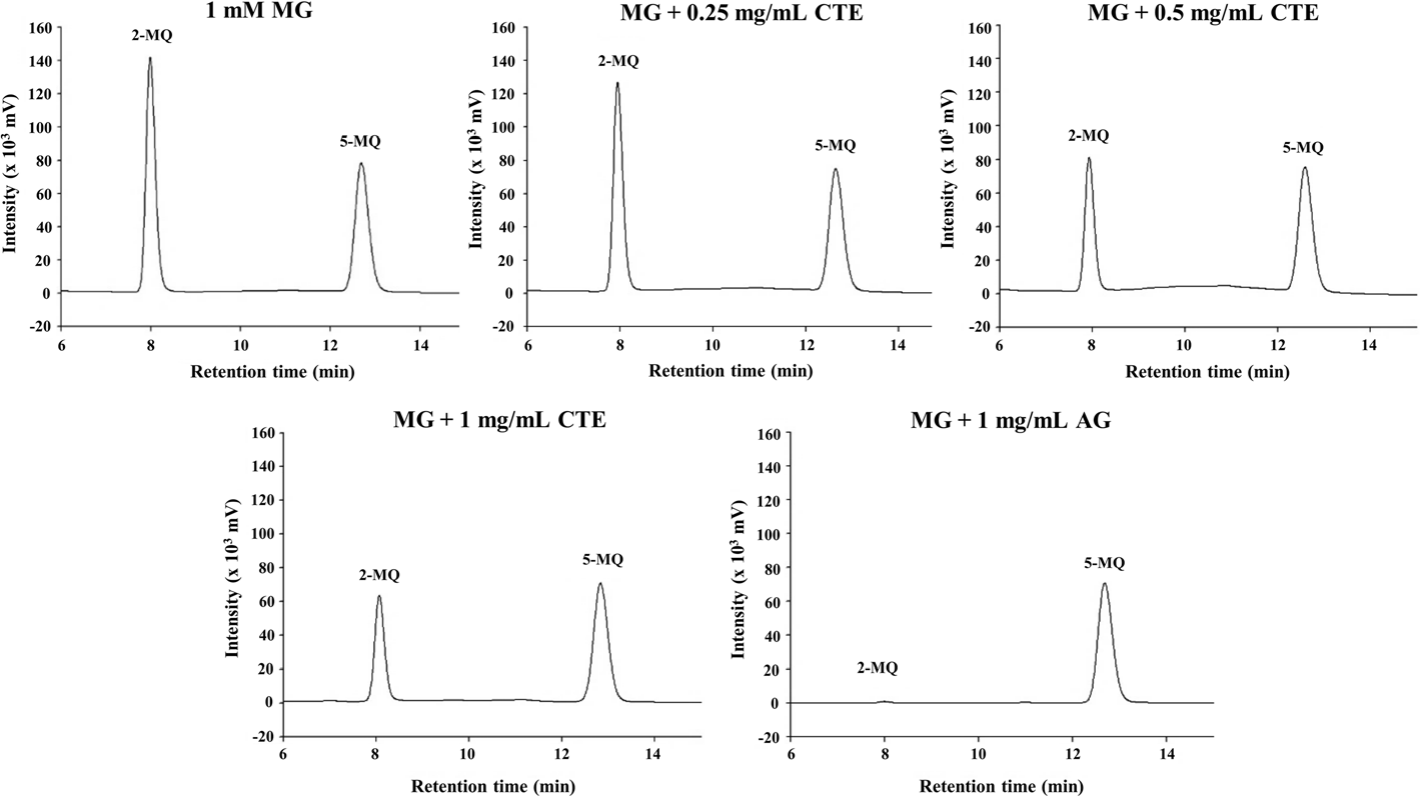
The HPLC chromatogram of methylglyoxal (MG) after reaction with *Clitoria ternatea* flower extract (CTE, 0.125–1 mg/mL) and aminoguanidine (AG, 1 mg/mL). MG was detected as 2-methylquinoxaline(2-MQ) after derivatization using *o*-phenylenediamine at 315 nm. 5-methylquinoxaline (5-MQ) was used as the internal standard

The level of 2-MQ represented free MG remaining after 24 h of incubation. The HPLC chromatogram showed a gradual reduction of 2-MQ peak as CTE concentration increased from 0.25 to 1 mg/mL (Fig. 5). The percentage of MG trapped by CTE was ranged from 15 to 43% (*p* < 0.05). In addition, the 2-MQ peak was flattened due to MG incubation with AG (1 mg/mL). This positive control had the ability to trap about 99% of MG.

## Discussion

Progression of macrovascular and microvascular diabetic complications has been associated with the formation of sugar-derived substances called advanced glycation end products (AGEs) via non-enzymatic glycation process [2]. AGE formation and accumulation is markedly accelerated in diabetes due to the elevation of available precursors such as glucose and fructose [2, 36]. In addition, it has been shown that methylglyoxal (MG) is one of the most important dicarbonyl compounds that irreversibly reacts with amino acids in protein molecules, lysine and arginine in particular, to form AGEs [37]. In 2008, Li and colleagues reported that MG was more reactive inducer than glucose to promote fluorescent AGE formation [38]. In this study, the formation of fluorescent AGEs in BSA mediated by MG was increased after 7 days. MG also induced an increase in carbonyl content and the reduction of free thiol groups, which are markers of oxidative damage to protein in glycated BSA. The current findings are in agreement with those reported by other authors, who demonstrated that MG induced protein glycation and caused oxidative damage to protein at the late stage of protein glycation [34, 39]. In this work, we demonstrated that CTE effectively inhibited dicarbonyl intermediate MG-derived AGEs formation and prevented protein oxidation. Our earlier investigations also revealed the anti-glycation property of CTE through the inhibition of fructose-induced glycated BSA during the initiation stage of glycation process associated with the amelioration of Amadori product fructosamine [30]. At the same concentrations as those adopted in the present study (0.25–1 mg/mL), CTE exhibited a greater potential than BSA/MG to inhibit AGE formation in the BSA/fructose system. Although the high CTE concentration up to 1 mg/mL were investigated for anti-glycation activity, no toxicity was observed in 3T3-L1 adipocytes at the concentration up to 2 mg/mL after 9 days of incubation [27]. Taken together, these findings indicated that CTE could prevent protein glycation and oxidation induced by both monosaccharide at the initiation stage and reactive dicarbonyl compound at the intermediate stage of protein glycation. Additional studies in diabetic or MG-treated animal models are nonetheless needed to investigate the efficacy of oral CTE supplementation as antiglycating agent.

Apart from causing glycation damage to protein, MG has been shown to contribute to oxidative DNA damage and strand breakage [13, 14]. During amino acids glycation by MG, free radicals including MG-protonated cation and MG-radical anion which are capable of donating electron to the oxygen molecule, causing the generation of superoxide anions and hydroxyl radicals [11, 40]. This process is highly accelerated in the presence of transition metal ions (such as copper and ferric ion) through the Fenton reaction to generate a greater number of hydroxyl radicals, which are apparently one of the most important ROS species involving in damage to DNA manifesting strand breakage [11, 13]. In the current study, CTE was shown to inhibit MG/lysine-induced oxidative DNA damage in the presence of copper ions. CTE also showed an ability to suppress the generation of both superoxide anion and hydroxyl radicals during the glycation process mediated by the interaction of MG and lysine. The mechanism by which CTE inhibits oxidative DNA damage is likely related to its free radical scavenging activity rather than metal ion chelating activity. This assertion is supported by the finding reported by Chayaratanasin et al. indicating the free radical scavenging activity of CTE. However, low potency of CTE to chelate metal ion studied by in vitro ferrous ion chelating power was noted [30]. In addition to oxidative process mediated by the glycation of MG and lysine, AAPH, a peroxyl radical generator, has also been widely used for inducing oxidative damage in lipids, proteins and DNA [41, 42]. In the present study, CTE was shown to prevent oxidative DNA damage induced by AAPH. These results are consistent with those reported by Phrueksanan et al. who demonstrated the preventive effect of CTE against AAPH-induced hemolysis and oxidative damage in erythrocytes [43]. Based on our findings, we can surmise that CTE is capable of scavenging different types of free radicals, thereby preventing the AGE formation and oxidative modification of proteins and DNA.

The current findings further indicate that CTE has the ability to directly trap MG, suggesting that carbonyl scavenging activity of CTE may be a mechanism responsible for the prevention of MG-induced AGE formation. The MG-trapping ability of CTE may also explain its action in preventing oxidative damage to proteins and DNA related to MG-mediated superoxide anion and hydroxyl radical production during glycation process. In previous studies, flavonoids identified in CTE were reported to have the ability to scavenge MG (Table S1). According to the supporting evidence, delphinidin derivatives and quercetin-3-rutinoside might be the active compounds in CTE that are responsible for MG-trapping ability, thus inhibiting of AGE formation [44, 45]. Shao et al. proposed the active site of flavonoids required for scavenging MG [46]. The A ring of flavonoid, C-6 and C-8 positions, is the active site for MG conjugation, and the hydroxyl group at C-5 enhances MG-trapping efficacy [46]. Therefore, the MG scavenging activity of CTE may result from the presence of flavonoids, especially delphinidin derivatives and flavonol glycosides, which contain available active sites on the A ring that influence the trapping capacity [27]. Further studies are thus needed to elucidate the structure of adducts from CTE-MG trapping reaction.

## Conclusions

**Fig. 6:**
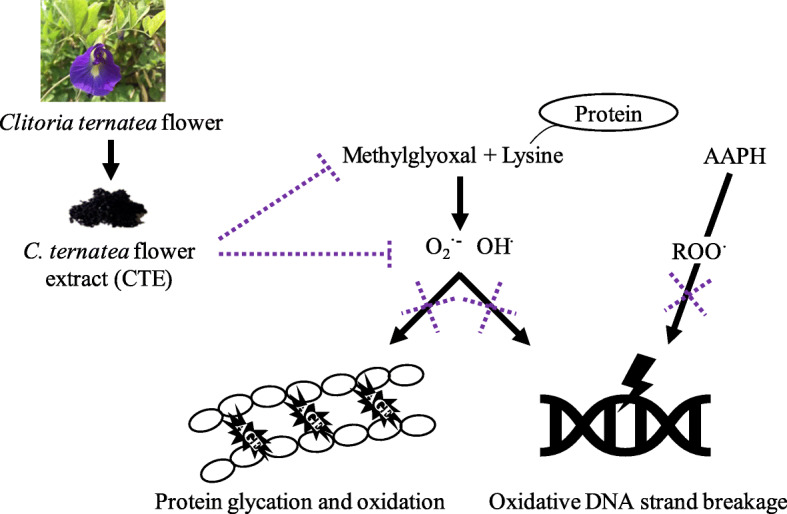
A Schematic illustration of underlying mechanisms for the inhibition of protein glycation and oxidative damage of protein and DNA by *Clitoria ternatea* L. flower extract (CTE)

CTE inhibited MG-induced protein glycation and oxidation-dependent damage to protein and DNA. It is suggested that the different types of free radical scavenging and carbonyl trapping ability may be the mechanism underlying these actions (Fig. 6). The study findings suggest that CTE might be a candidate plant extract for the prevention of MG-induced glycation and oxidative damage.

## Supplementary Information

**Additional file 1: Table S1.** Methylglyoxal (MG)-trapping ability of the phytochemical compounds identified in *Clitoria ternatea* L. flower extract (CTE) from the previous study (1).

## Data Availability

The datasets used and/or analyzed during the current study available from the corresponding author Dr. Thavaree Thilavech (Thavaree.thi@mahidol.ac.th) on reasonable request.

## Abbreviations

CTE: *Clitoria ternatea* L. flower extract
MG: Methylglyoxal
AGE: Advanced glycation end products
AAPH: 2,2′-azobis(2-methylpropionamidine)dihydrochloride
AG: Aminoguanidine
TBARS: Thiobarbituric acid reactive substances
SC: Supercoiled
OC: Open circular
2-MQ: 2-methylquinoxaline
