# Supplementary material for: Protective role of Clitoria ternatea L. flower extract on methylglyoxal-induced protein glycation and oxidative damage to DNA
Source: BMC Complement Med Ther. 2021 Mar 1;21:80. doi: 10.1186/s12906-021-03255-9 (PMC7923514; doi:10.1186/s12906-021-03255-9)
Supplement: Supplementary file 1 — Additional file 1: Table S1. Methylglyoxal (MG)-trapping ability of the phytochemical compounds identified in Clitoria ternatea L. flower extract (CTE) from the previous study (1). [file 12906_2021_3255_MOESM1_ESM.docx]

**Supplementary material**

**Table S1.** Methylglyoxal (MG)-trapping ability of the phytochemical compounds identified in *Clitoria ternatea* L. flower extract (CTE) from the previous study (1)

| **No.** | **Compounds** | **Methylglyoxal (MG)-trapping ability** | **References** |
| --- | --- | --- | --- |
|  | Delphinidin derivatives | - Isolated delphinidin-3-rutinoside from blackcurrant was able to trap MG, and yield anthocyanin-mono-MG adduct. - The bilberry delphinidin derivatives including delphinidin 3-O-galactoside, delphinidin 3-O-glucoside and delphinidin 3-O-arabinoside exerted noticeable and comparable MG trapping ability which was supported by the reduction of each delphinidin derivatives in the reaction mixture of bilberry extract together with MG. | (2)  (3) |
|  | Delphinidin-3-glucoside (myrtillin) |  |  |
|  | Delphinidin-3-*O*-(6-O-β-coumaryl)glucoside-puruvic acid |  |  |
|  | Preternatin A3 | - There is an almost complete lack of data in the literature for preternatin and ternatins as MG-trapping agents. |  |
|  | Ternatin D1 |  |  |
|  | Ternatin D2 |  |  |
|  | Ternatin B2 |  |  |
|  | Kaemferol-3-*O*-rutinoside | - The mono-MG and di-MG adducts of kaempferol were identified. - The antiglycation activity of kaemferol-3-O-rutinoside has been reported in glucose/bovine serum albumin (BSA) system. However, the MG-trapping ability of kaemferol-3-O-rutinoside has never been explored. | (4)  (5) |
|  | Kaemferol-3-*O*-(2-rhamnosyl)rutinoside |  |  |
|  | (+)-Catechin 7-O-β-glucoside | - (+)-Catechin trapped MG in a time- and concentration-dependent manner and formed mono-MG and di-MG adducts. However, no study has been conducted to investigate the role of catechin glycoside derivatives on antiglycation and MG-trapping action. | (6) |
|  | Syringetin-3-*O*-glucoside | - There has no report for syringetin and its derivatives as MG-trapping and antiglycating agent. |  |
|  | Quercetin-3-rutinoside (rutin) | - Quercetin-3-rutinoside exhibited the ability to trap MG by forming mono-MG and di-MG adducts. | (7) |
|  | Quercetin triglycoside | - Di-MG adducts of quercetin was identified as the major products whereas the lower amounts of mono-MG adducts of quercetin was found. The results were in agreement the inhibitory activity quercetin on the formation of advanced glycation end products in MG/BSA system. Although quercetin triglycoside is a derivative of quercetin, there is no report for quercetin triglycoside as MG-trapping or antiglycating agent | (8) |

**References**

1. Chayaratanasin P, Caobi A, Suparpprom C, Saenset S, Pasukamonset P, Suanpairintr N, et al., Clitoria ternatea flower petal extract inhibits adipogenesis and lipid accumulation in 3T3-L1 preadipocytes by downregulating adipogenic gene expression. Molecules. 2019;24(10):1894.
2. Chen X-Y, Huang I-M, Hwang LS, Ho C-T, Li S, Lo C-Y. Anthocyanins in blackcurrant effectively prevent the formation of advanced glycation end products by trapping methylglyoxal. J Funct Foods. 2014;8:259-68. https://doi.org/10.1016/j.jff.2014.03.025.
3. Fraisse D, Bred A, Felgines C, Senejoux F. Screening and characterization of antiglycoxidant anthocyanins from Vaccinium myrtillus Fruit using DPPH and methylglyoxal pre-column HPLC assays. Antioxidants. 2020;9(6):512.
4. Yang BN, Choi EH, Shim SM. Inhibitory activities of kaempferol against methylglyoxal formation, intermediate of advanced glycation end products. Appl Biol Chem. 2017;60(1):57-62.
5. Lal Shyaula S, Abbas G, Siddiqui H, A Sattar S, Iqbal Choudhary M, Z Basha F. Synthesis and antiglycation activity of kaempferol-3-O-rutinoside (nicotiflorin). Med Chem. 2012;8(3):415-20.
6. Zhu D, Wang L, Zhou Q, Yan S, Li Z, Sheng J, Zhang W. (+)‐Catechin ameliorates diabetic nephropathy by trapping methylglyoxal in type 2 diabetic mice. Mol Nutr Food Res. 2014c;58(12):2249-60.
7. Bednarska K, Kuś P, Fecka I. Investigation of the phytochemical composition, antioxidant activity, and methylglyoxal trapping effect of Galega officinalis L. Herb in vitro. Molecules. 2020;25(24):5810.
8. Li X, Zheng T, Sang S, Lv L. Quercetin inhibits advanced glycation end product formation by trapping methylglyoxal and glyoxal. J Agric Food Chem. 2014;62(50):12152-8.

**
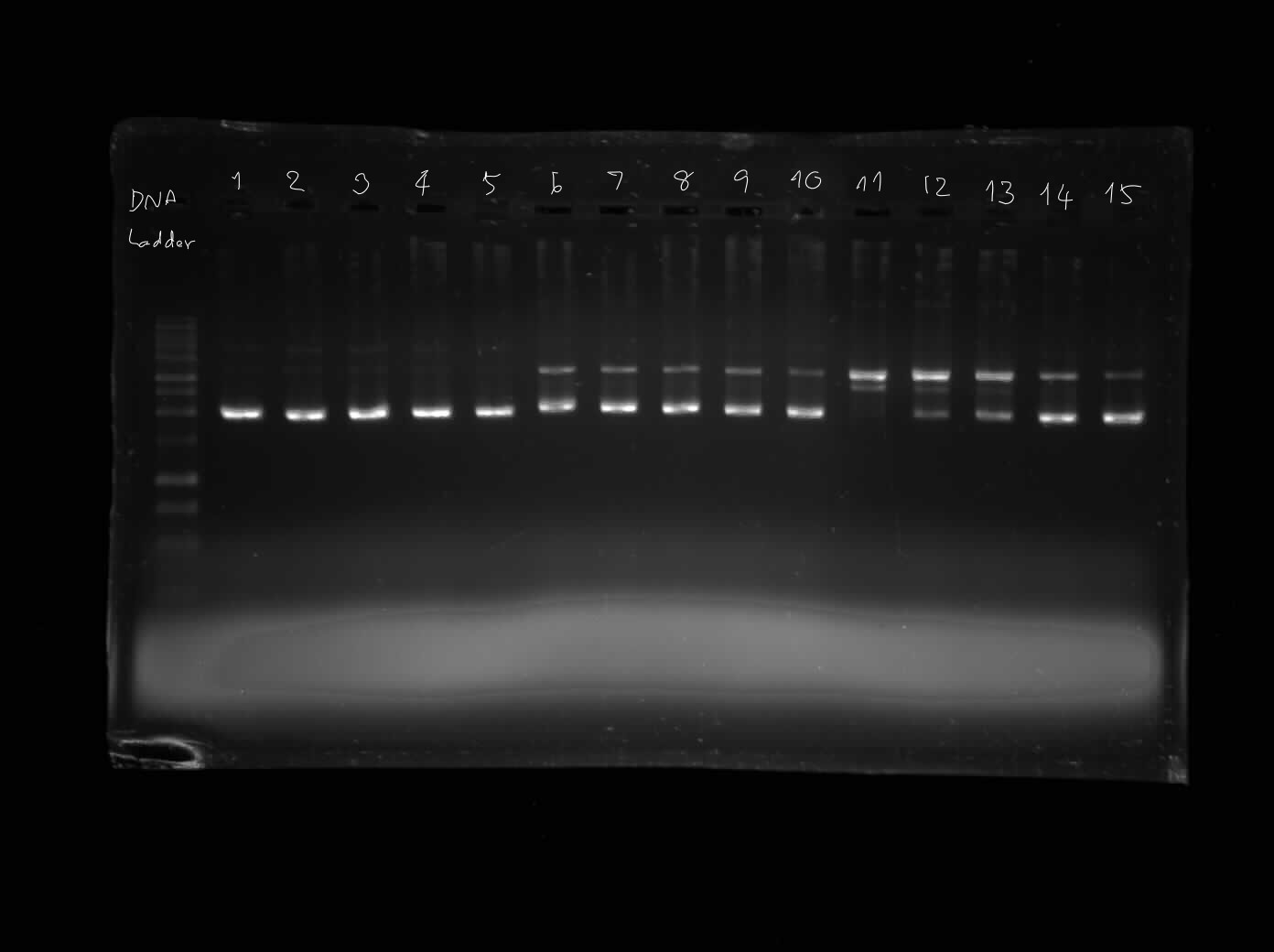
**

**Fig. 3 (uncropped)** Effect of *Clitoria ternatea* flower extract (CTE) on DNA strand breakage in methylglyoxal (MG)/lysine and 2,2′-azobis(2-methylpropionamidine) dihydrochloride (AAPH) system. Supercoiled form (SC) and opened circular form (OC) represented as the major band and damaged plasmid DNA, respectively. Plasmid DNA (0.25 μg) was incubated with the following: Lane 1, DNA alone; Lane 2, 50 mM MG; Lane 3, 50 mM lysine; Lane 4, 0.25 mg/mL CTE; Lane 5, 1 mg/mL CTE; Lane 6, MG+lysine; Lane 7, MG+lysine+Cu^2+^; Lane 8, MG+lysine+Cu^2+^+0.25 mg/mL CTE; Lane 9, MG+lysine+Cu^2+^+0.5 mg/mL CTE; Lane 10, MG+lysine+Cu^2+^+1 mg/mL CTE; Lane 11, 12.5 mM AAPH; Lane 12, AAPH+0.125 mg/mL CTE; Lane 13, AAPH+0.25 mg/mL CTE; Lane 14, AAPH+0.5 mg/mL CTE; Lane 15, AAPH+1 mg/mL CTE.
